# The Humanistic Burden of Small Cell Lung Cancer (SCLC): A Systematic Review of Health-Related Quality of Life (HRQoL) Literature

**DOI:** 10.3389/fphar.2017.00339

**Published:** 2017-06-15

**Authors:** Bryan M. Bennett, Jane R. Wells, Charlotte Panter, Yong Yuan, John R. Penrod

**Affiliations:** ^1^Adelphi Values, Patient-Centered OutcomesBollington, United Kingdom; ^2^Health Economics and Outcomes Research, Bristol-Myers SquibbPrinceton, NJ, United States

**Keywords:** small cell lung carcinoma, systematic literature review, quality of life, outcome assessment (health care), surveys and questionnaires, activities of daily living, health impact assessment

## Abstract

**Background:** Little is known about the humanistic burden of small cell lung cancer (SCLC), specifically the impact on health-related quality of life (HRQoL). The aim of this systematic literature review was to explore the impact of SCLC on HRQoL and the patient reported outcomes (PROs) used to capture this impact.

**Methods:** We conducted a systematic search of Medline®, Embase, and PsycINFO, oncology organization websites and conference proceedings within the past 10 years. Articles reporting HRQoL outcomes of SCLC patients were selected.

**Results:** Twenty-seven eligible publications were identified. Global or overall impact on HRQoL (*n* = 21) was reported most often, with considerably fewer reporting individual domains that comprise HRQoL. Results indicated that HRQoL was negatively impacted in SCLC patients in comparison to the normal population in most domains. Overall, the domains measuring physical functioning and activities of daily living were most impacted. However, results on cognitive and emotional functioning were inconclusive. The impact on HRQoL may be least in both limited disease and extensive disease (ED) SCLC patients who have responded to treatment, and greatest in ED patients who were treatment naïve. The most frequently used PROs were the EORTC QLQ-C30 core cancer instruments, the lung cancer specific module the EORTC QLQ-LC13, LCSS, and EQ-5D.

**Conclusion:** There exists a paucity of reporting on SCLC HRQoL outcomes. This extends to the reporting of domain level scores and by patient sub-group. Greater reporting at a granular level is recommended to allow for more robust conclusions to be made.

## Introduction

Lung cancer is the second most prevalent form of cancer with more than 1.8 million new cases being diagnosed annually (Siegel et al., 2015). It is the leading cause of cancer mortality, being responsible for 19.4% of all cancer related deaths (World Health Organization, 2012). Approximately 15% of lung cancer cases are small cell lung cancer (SCLC), and cigarette smoking is a dominant risk factor of SCLC, accounting for 95% of SCLC (Zikos et al., 2014). Some key characteristics of SCLC, such as rapid progression and earlier metastasis (World Health Organization, 2012), differentiate it from the other forms of epithelial lung cancers known as non-small cell lung cancer (NSCLC). Combined with the fact that diagnoses often occurs in the later stage due to the non-specific symptoms, SCLC has a poor prognosis (Gorman, 2012). The median survival following diagnosis is 16–24 and 6–12 months for limited-stage and extensive-stage, respectively. Further, the 5 year survival rate is just 5–10%. Thus, SCLC represents considerable disease burden, with significant impact on survival and deterioration of health-related quality of life (HRQoL) (Henry et al., 2008).

In comparison to SCLC, there is a greater body of research measuring the humanistic burden of NSCLC and the impact this has on HRQoL, with findings indicating a considerable burden (Enstone et al., 2015). A consistent trend in the NSCLC HRQoL literature reports deterioration in the emotional, physical, social, cognitive domains as well as activities of daily living (Enstone et al., 2015). A key impact identified was impairment in physical functioning, with NSCLC patients reporting reduced physical functioning as their disease progresses. Given the differentiation in some key characteristics of SCLC vs. the greater majority of lung cancers, the impact on quality of life may differ as well.

The objective of this systematic literature review (SLR) was to complete a robust and comprehensive review of the impact of SCLC on HRQoL. A further objective of this review was to identify the PROs used to measure HRQoL in SCLC. How the impact of SCLC differs within its patient sub-populations, including disease stage (limited [LD] and extensive [ED]), line of therapy, PD1/PD-L1 expression, smoking history and status, and the presence of brain metastases, was also examined.

## Methodology

A literature search was conducted, in line with Cochrane Handbook for Systematic Reviews of Interventions (CHSRI) guidelines (Higgins and Green, 2005), to identify publications reporting on the impact of SCLC on HRQoL. The following computerized bibliographic databases were searched using the OVID search engine for the SLR: PUBMED (Medline), Medline® In-Process, Embase, and PsycINFO. The search was limited to studies published in the past 10 years (1st January 2005–24th February 2016). The search utilized a combination of disease and HRQoL impact subject headings and free text searching to ensure that the most relevant literature was identified (see Table 1 for search strategy).

**Table 1:** Search strategy.

	**Search terms**	**Results**
	Small cell lung carcinoma OR carcinoma, small cell OR SCLC OR small cell lung cancer OR small-cell lung cancer OR small cell carcinoma OR small-cell carcinoma OR small cell undifferentiated carcinoma OR small-cell undifferentiated carcinoma OR oat cell carcinoma OR oat-cell carcinoma OR combined cell carcinoma	28,606
AND		
	(Health-related adj1 quality adj1 of adj1 life) OR (Quality adj1 of adj1 life) OR (patient adj1 burden) OR (patient adj1 impact) OR (burden adj1 of adj1 illness) OR (activities adj1 of adj1 daily adj1 living) OR (daily adj1 activities) OR (psychological adj1 function$) OR (social adj1 function$) OR (emotional adj1 function$) OR (physical adj1 function$) OR HRQOL OR QOL OR work OR (symptom adj1 burden) OR (symptom adj1 assessment) OR quality of life	2,465,885
Total		751
Limit to human/humans		752
Limit to 2005-current		411
Total records identified (duplicates removed)		373

All abstracts identified in the search were systematically screened against eligibility criteria for full-publication review by two independent reviewers. Any disagreement was resolved by a third senior researcher. Publications reporting HRQoL data associated with lung cancer populations without specific reference to SCLC, or reporting data on treatment efficacy/interventional data in SCLC that did not assess HRQoL (or specific domains of HRQoL) were excluded. Publications consisting of case studies, letters, editorials, and commentaries were also excluded. Only publications reporting data or findings specifically concerning overall or global HRQoL or domains of HRQoL of patients with SCLC were included in the full-text review.

A search of the gray literature included conference proceedings from the annual European, US, Asia-Pacific, and Latin American congresses of the International Society for Pharmacoeconomics and Outcomes Research (ISPOR), the American Society of Clinical Oncology (ASCO), the European Society for Medical Oncology (ESMO), the annual European Cancer Congress (ECC), and the annual World Conference on Lung Cancer (WCLC).

**Figure 1:**
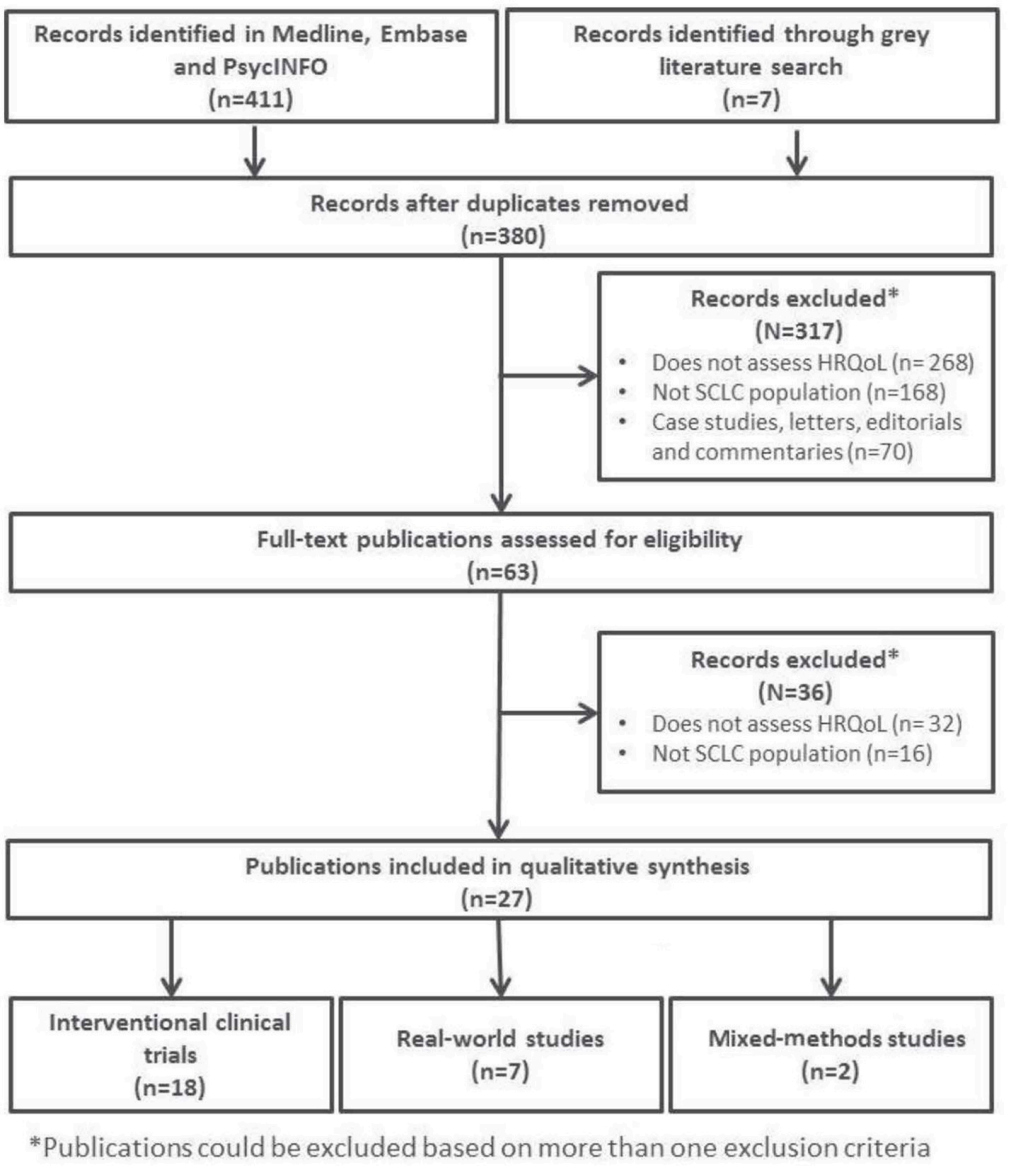
PRISMA flow chart of included and excluded articles.

Figure 1 provides a schematic overview of the included articles for full-text review.

## Results

The 27 publications included in this review reported results from interventional clinical trials assessing HRQoL as an endpoint (*n* = 18); 11 were randomized but not controlled (Thatcher et al., 2005; Eckardt et al., 2006; Reck et al., 2006, 2012; Hermes et al., 2008; Lee et al., 2009; Le Pechoux et al., 2011; Nagel et al., 2011; Wolfson et al., 2011; Satouchi et al., 2014; Sekine et al., 2014) (see Table 2), four were randomized controlled trials (RCTs) (Giaccone et al., 2005; O'Brien et al., 2006; Bottomley et al., 2008; Slotman et al., 2009) (see Table 3) and three were non-randomized interventional studies (Fennell et al., 2007; Araujo et al., 2009; Eckardt et al., 2009) (see Table 4). The remaining publications were real world (*n* = 7) (Jatoi et al., 2005; Chen et al., 2007, 2008, 2012; Rolke et al., 2008; Kohli et al., 2010; Nakahara et al., 2015) (see Table 5) and mixed-methods studies (*n* = 2) (Westerman et al., 2007, 2008) (see Table 6).

**Table 2:** Randomized trials (no control group) included in the SLR.

**References**	**Title**	**Disease stage**	**Line of therapy at baseline/observation**	**Response to treatment at baseline/ observation**	**Patient-reported outcomes used**	**Domains of HRQoL reported**					
						**Gbl/OA**	**ADL**	**EF**	**PF**	**SF**	**CF**
Eckardt et al., 2009	Phase II study of picoplatin as second-line therapy for patients with small-cell lung cancer	23% LD, 77% ED	1st line	Experienced disease progression	LCSS	X	–	–	–	–	–
Hermes et al., 2008	Irinotecan plus carboplatin vs. oral etoposide plus carboplatin in extensive small-cell lung cancer: a randomized phase III trial	100% ED	Systemic treatment-naïve	Not applicable	EORTC QLQ-C30, EORTC QLQ-LC13	X	X	X	X	X	X
Lee et al., 2009	Anti-angiogenic therapy using thalidomide combined with chemotherapy in small cell lung cancer: a randomized, double-blind, placebo-controlled trial	51% LD, 49% ED	Chemotherapy-naïve	Not applicable	EORTC QLQ-C30, EORTC QLQ-LC13, LLCG DDC	X	X	X	X	X	X
Le Pechoux et al., 2011	Clinical neurological outcome and quality of life among patients with limited small-cell cancer treated with two different doses of prophylactic cranial irradiation in the intergroup phase III trial (PCI99-01, EORTC 22003-08004, RTOG 0212, and IFCT 99-01)	100% LD	> 1st line	Complete remission	EORTC QLQ-C30, EORTC QLQ-BN20	X	X	X	X	X	X
Nagel et al., 2011	Addition of darbepoetin alfa to dose-dense chemotherapy: results from a randomized phase II trial in small-cell lung cancer patients receiving carboplatin plus etoposide	41.7, 44.4% LD (group 1, group 2), 58.3% + 55.6% ED (group 1, group 2)	Chemotherapy-naïve	Not applicable	EORTC QLQ-C30	X	X	X	X	X	X
Reck et al., 2012	Baseline quality of life and performance status as prognostic factors in patients with extensive-stage disease small cell lung cancer treated with pemetrexed plus carboplatin vs. etoposide plus carboplatin	100% ED	Chemotherapy-naive	Not applicable	FACT-G	X	X	X	X	X	–
Reck et al., 2006	Efficient palliation in patients with small-cell lung cancer by a combination of paclitaxel, etoposide, and carboplatin: quality of life and 6-years'-follow-up results from a randomized phase III trial	50% LD, 50% ED	Treatment-naïve	Not applicable	EORTC QLQ-C30	X	X	X	X	X	X
Satouchi et al., 2014	Phase III study comparing amrubicin plus cisplatin with irinotecan plus cisplatin in the treatment of extensive-disease small-cell lung cancer: JCOG 0509	100% ED	Chemotherapy naïve	Not applicable	QOL-ACD, EORTC QLQ-C30	–	–	–	X	–	–
Sekine et al., 2014	A randomized phase III study of single-agent amrubicin vs. carboplatin/etoposide in elderly patients with extensive-disease small-cell lung cancer	100% ED	Systemic chemotherapy-naïve	Not applicable	FACT-L, EQ-5D	X	–	–	–	–	–
Thatcher et al., 2005	Ifosfamide, carboplatin, and etoposide with midcycle vincristine vs. standard chemotherapy in patients with small-cell lung cancer and good performance status: clinical and quality-of-life results of the British Medical Research Council multicenter randomized LU21 trial	85, 86% LD (group 1, group 2), 15, 14% ED (group 1, group 2), 3% Uncertain	Treatment naïve	Responded to therapy	EORTC QLQ-C30, EORTC QLQ-LC, HADS, RSCL—modified to include additional lung cancer specific items	X	X	X	–	–	–
Wolfson et al., 2011	Primary analysis of a phase II randomized trial Radiation Therapy Oncology Group (RTOG) 0212: impact of different total doses and schedules of prophylactic cranial irradiation on chronic neurotoxicity and quality of life for patients with limited-disease small-cell lung cancer	100% LD	>1st line	Complete response	EORTC QLQ-C30, EORTC QLQ-BN20	X	–	–	–	–	X
TOTAL						10	7	7	7	7	6

*LD, Limited disease; ED, Extensive disease; HRQoL, Health-related quality of life; Gbl/OA, Global/Overall; ADL, Activities of daily living; EF, Emotional functioning; PF, Physical functioning; SF, Social functioning; CF, Cognitive functioning; EORTC QLQ-C30, European Organization for Research and Treatment of Cancer Quality of Life Core 30 Questionnaire; EORTC QLQ-LC, Lung cancer specific module; EORTC QLQ-BN20, Brain cancer specific module; FACT-G, Functional Assessment of Cancer Therapy-General; FACT-L, Functional Assessment of Cancer Therapy-Lung cancer; LCSS, Lung Cancer Symptom Scale, RSCL, Rotterdam Symptom Checklist; QOL-ACD, Questionnaire for patients with cancer treater with anticancer drugs*.

**Table 3:** Randomized controlled trials (RCT) included in the SLR.

**References**	**Title**	**Disease stage**	**Line of therapy at baseline/observation**	**Response to treatment at baseline/ observation**	**Patient-reported outcomes used**	**Domains of HRQoL reported**					
						**Gbl/OA**	**ADL**	**EF**	**PF**	**SF**	**CF**
Bottomley et al., 2008	Symptom and quality of life results of an international randomized phase III study of adjuvant vaccination with Bec2/BCG in responding patients with limited disease small-cell lung cancer	100% LD	> 2nd line	Major response (partial or complete)	EORTC QLQ-C30, EORTC QLQ-LC13	X	X	X	X	X	X
Giaccone et al., 2005	Phase III study of adjuvant vaccination with Bec2/bacille Calmette-Guerin in responding patients with limited-disease small-cell lung cancer (European Organization for Research and Treatment of Cancer 08971-08971B; Silva Study)	100% LD	>2nd line	Major response (partial or complete)	EORTC QLQ-C30, EORTC QLQ-LC13	X	–	–	–	–	–
O'Brien et al., 2006	Phase III trial comparing supportive care alone with supportive care with oral topotecan in patients with relapsed small-cell lung cancer	39, 32% LD (group 1, group 2), 61, 68% ED (group 1, group 2)	1st line	Relapsed	EQ-5D, PSA	X	X	–	–	–	–
Slotman et al., 2009	Prophylactic cranial irradiation in extensive disease small-cell lung cancer: short-term health-related quality of life and patient reported symptoms: results of an international Phase III randomized controlled trial by the EORTC Radiation Oncology and Lung Cancer Groups	100% ED	>1st line	Responded to chemotherapy	EORTC QLQ-C30, EORTC QLQ-BN20	X	X	X	X	X	X
TOTAL						4	3	2	2	2	2

*LD, Limited disease; ED, Extensive disease, HRQoL, Health-related quality of life; Gbl/OA, Global/Overall; ADL, Activities of daily living; EF, Emotional functioning; PF, Physical functioning; SF, Social functioning; CF, Cognitive functioning; EORTC QLQ-C30, European Organization for Research and Treatment of Cancer Quality of Life Core 30 Questionnaire; EORTC QLQ-LC, Lung cancer specific module; EORTC QLQ-BN20, Brain cancer specific module; EQ-5D, EuroQol 5-Dimensions Health Questionnaire; PSA, Patient Self-Assessment*.

**Table 4:** Non-randomized interventional studies (single arm trials) included in the SLR.

**References**	**Title**	**Disease stage**	**Line of therapy at baseline/ observation**	**Response to treatment at baseline/observation**	**Patient-reported outcomes used**	**Domains of HRQoL reported**					
						**Gbl/OA**	**ADL**	**EF**	**PF**	**SF**	**CF**
Araujo et al., 2009	Phase II study of celecoxib with cisplatin plus etoposide in extensive-stage small cell lung cancer	100% ED	Chemotherapy-naïve	Not applicable	EORTC QLQ-C30, EORTC QLQ-LC13	X	X	X	X	X	X
Eckardt et al., 2009	Phase II study of picoplatin as second-line therapy for patients with small-cell lung cancer	23% LD, 77% ED	1st line	Experienced disease progression	LCSS	X	–	–	–	–	–
Fennell et al., 2007	Phase II trial of irinotecan, cisplatin and mitomycin for relapsed small cell lung cancer	81% LD, 19% ED	1st line	Relapsed	RSCL	X	X	X	X	X	X
TOTAL						3	2	2	2	2	2

*LD, Limited disease; ED, Extensive disease; HRQoL, Health-related quality of life; Gbl/OA, Global/Overall; ADL, Activities of daily living; EF, Emotional functioning; PF, Physical functioning; SF, Social functioning; CF, Cognitive functioning; EORTC QLQ-C30, European Organization for Research and Treatment of Cancer Quality of Life Core 30 Questionnaire; EORTC QLQ-LC, Lung cancer specific module; LCSS, Lung Cancer Symptom Scale; RSCL, Rotterdam Symptom Checklist*.

**Table 5:** Real-world studies included in the SLR.

**References**	**Title**	**Disease stage**	**Line of therapy at baseline/observation**	**Response to treatment at baseline/observation**	**Patient-reported outcomes used**	**Domains of HRQoL reported**					
						**Gbl/OA**	**ADL**	**EF**	**PF**	**SF**	**CF**
Chen et al., 2012	Effect of cigarette smoking on quality of life in small cell lung cancer patients	38% ED	Not reported (Cohort study)	Not reported (Cohort study)	LCSS, LASA	X	–	–	–	–	–
Chen et al., 2008	Symptom assessment in relapsed small cell lung cancer: cross-validation of the patient symptom assessment in lung cancer instrument	39, 32% LD (group 1, group 2), 61, 68% ED (group 1, group 2)	1st line	Relapsed	EQ-5D and PSALC	–	X	–	–	–	–
Chen et al., 2007	Psychometric validation of the Patient Symptom Assessment in Lung Cancer instrument for small cell lung cancer	16.8, 15.4% LD (group 1, group 2), 83.2, 84.6% ED (group 1, group 2)	1st line	Relapsed	PSALC	–	X	–	–	–	–
Jatoi et al., 2005	Exploring Vitamin and Mineral Supplementation and Purported Clinical Effects in Patients With Small Cell Lung Cancer: Results From the Mayo Clinic Lung Cancer Cohort	60% LD, 40% ED	Not reported (Cohort study)	Not reported (Cohort study)	LCSS	X	–	–	–	–	–
Kohli et al., 2010	Cancer treatment impact on cognitive functioning and quality of life in lung cancer survivors	LD and ED	Not reported (Cohort study)	Not reported (Cohort study)	LCSS, LASA	–	–	–	–	–	X
Nakahara et al., 2015	Neurotoxicity due to prophylactic cranial irradiation for small-cell lung cancer: A retrospective analysis	100% LD	>1st line	Complete and partial response	HDS-R	–	–	–	–	–	X
Rolke et al., 2008	Health related quality of life, mood disorders and coping abilities in an unselected sample of patients with primary lung cancer	50% LD, 50% ED	Treatment-naïve	Not applicable	EORTC QLQ-C30, HADS, SoC 13	X	X	X	X	X	X
TOTAL						3	3	1	1	1	3

*LD, Limited disease; ED, Extensive disease; HRQoL, Health-related quality of life; Gbl/OA, Global/Overall; ADL, Activities of daily living; EF, Emotional functioning; PF, Physical functioning; SF, Social functioning; CF, Cognitive functioning; EORTC QLQ-C30, European Organization for Research and Treatment of Cancer Quality of Life Core 30 Questionnaire; EQ-5D, EuroQol 5-Dimensions Health Questionnaire; HADS, Hospital Anxiety and Depression Scale; HDS-R, Hasegawa Dementia Scale-Revised; LASA, Linear Analog Self-Assessment; LCSS, Lung Cancer Symptom Scale; PSALC, Patient Symptom Assessment in Lung Cancer; SoC 13, Sense of Coherence 13*.

**Table 6:** Mixed-methods studies included in the SLR.

**References**	**Title**	**Disease stage**	**Line of therapy at baseline/ observation**	**Response to treatment at baseline/observation**	**Patient-reported outcomes used**	**Domains of HRQoL reported**					
						**Gbl/OA**	**ADL**	**EF**	**PF**	**SF**	**CF**
Westerman et al., 2008	Listen to their answers! Response behavior in the measurement of physical and role functioning	52% LD, 47.8% ED	Treatment-naïve	Not applicable	EORTC QLQ-C30, EORTC QLQ-LC13	–	–	X	–	–	–
Westerman et al., 2007	Small-cell lung cancer patients are just “a little bit” tired: response shift and self-presentation in the measurement of fatigue	52% LD, 47.8% ED	Treatment-naïve	Not applicable	EORTC QLQ-C30, EORTC QLQ-LC13, SEIQoL-DW	X	X	–	X	–	–
TOTAL						1	1	1	1	0	0

*LD, Limited disease; ED, Extensive disease; HRQoL, Health-related quality of life; Gbl/OA, Global/Overall; ADL, Activities of daily living; EF, Emotional functioning; PF, Physical functioning; SF, Social functioning; CF, Cognitive functioning; EORTC QLQ-C30, European Organization for Research and Treatment of Cancer Quality of Life Core 30 Questionnaire; EORTC QLQ-LC, Lung cancer specific module; SEIQoL-DW, Schedule for the evaluation of individual quality of life-Direct Weighting*.

### PROs used to assess HRQoL

**Figure 2:**
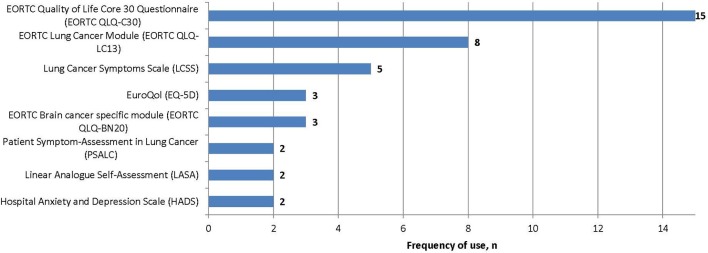
PROs used in identified SCLC literature and frequency of use.

Several PROs were identified in this review for capturing the humanistic burden of SCLC. The most commonly used PROs assessing HRQoL and symptoms were: the European Organization for Research and Treatment in Cancer Quality of Life Core 30 Questionnaire (EORTC QLQ-C30; *n* = 15), the Lung Cancer specific module (EORTC QLQ-LC13; *n* = 8), which is designed to be administered alongside the EORTC QLQ-C30, and the Lung Cancer Symptom Scale (LCSS; *n* = 5). Instruments identified only once include: London Lung Cancer Group (LLCG) Daily Diary Card (DDC), Functional Assessment of Cancer Therapy- General (FACT-G), Functional Assessment of Cancer Therapy- Lung (FACT-L), Questionnaire for patients with cancer treated with anticancer Drugs (QOL-ACD), Rotterdam Symptom Checklist (RSCL) modified to include additional lung cancer specific items, Rotterdam Symptom Checklist (RSCL), Hasegawa Dementia Scale-Revised (HDS-R), Sense of Coherence Questionnaire (SoC), Schedule for the evaluation of individual quality of life-direct weighting (SEIQoL-DW), and Patient Self-Assessment (PSA). See Figure 2 for a list of the most frequently used PROs identified and their frequency of use.

### Impact of SCLC on HRQoL

The majority of publications reported global or overall HRQoL (*n* = 21, 78%), with considerably fewer reporting specific domains of HRQoL: activities of daily living (ADL; *n* = 16, 59%), emotional functioning (EF; *n* = 14, 52%), cognitive functioning (CF; *n* = 13, 48%), physical functioning (PF; *n* = 13, 48%), and social functioning (SF; *n* = 11, 41%).

### Global HRQoL

Global HRQoL (Giaccone et al., 2005; Jatoi et al., 2005; Thatcher et al., 2005; Eckardt et al., 2006, 2009; O'Brien et al., 2006; Reck et al., 2006, 2012; Fennell et al., 2007; Bottomley et al., 2008; Hermes et al., 2008; Rolke et al., 2008; Westerman et al., 2008; Araujo et al., 2009; Lee et al., 2009; Slotman et al., 2009; Le Pechoux et al., 2011; Nagel et al., 2011; Wolfson et al., 2011; Chen et al., 2012; Sekine et al., 2014) was most frequently measured using two items (overall health and overall QoL) in the EORTC QLQ-C30 (*n* = 13, 62%). In the LCSS, the second most frequently used PRO to measure the concept (*n* = 4, 19%, Jatoi et al., 2005; Eckardt et al., 2006, 2009; Chen et al., 2012), global QoL is assessed via a single item.

Most chemotherapy naïve LD and ED SCLC patients had EORTC QLQ-C30 Global health/quality of life mean baseline scores ranged between 44.7 and 55.4 (Reck et al., 2006; Hermes et al., 2008; Rolke et al., 2008; Lee et al., 2009). One study however reported baseline scores that were over 10 points higher, at 66 points. The main difference with this study was that patients provided their responses during an interview, rather than completing the questionnaire in the clinical site (Westerman et al., 2008). SCLC patients (both ED and LD) who had responded to treatment (67, Bottomley et al., 2008; Slotman et al., 2009) reported higher scores than patients who had not been treated. Systemic treatment naïve ED SCLC patients had the lowest scores overall (44.7, Hermes et al., 2008). As expected, all SCLC EORTC QLQ-C30 scores were lower the normative global health status/QoL scores provided by the EORTC of 71.2 (Scott et al., 2008). Real world EORTC QLQ-C30 data could be drawn from one study only, reporting a mean score of 49.6 (Rolke et al., 2008). This score falls within the range of clinical trial EORTC QLQ-C30 baseline scores (Reck et al., 2006; Bottomley et al., 2008; Hermes et al., 2008; Lee et al., 2009; Slotman et al., 2009), indicating no difference in scores between the population types.

A significant difference in HRQoL was reported between a cohort of LD and ED SCLC patients and matched controls free of lung cancer, using a combined LCSS and Linear Analog Self-Assessment (LASA) score (63.4 vs. 80.9, *p* < 0.0001) (Chen et al., 2012). Examining smoking behavior, the authors reported that SCLC patients who were “early quitters” and “never smokers” enjoy a better overall HRQoL than those who never quit or quit post-diagnosis. However, smokers who quit post-diagnosis also experienced a positive impact on HRQoL (Chen et al., 2012). This publication was the only SCLC study to examine the impact of smoking status on HRQoL. While three publications (out of all publications included in this review) included PD1/PD-L1 expression in their patient sample, (Giaccone et al., 2005; Bottomley et al., 2008; Eckardt et al., 2009) no publications examined global health status/QoL scores by PD1/PD-L1 expression.

The literature revealed a trend for global/overall HRQoL scores remaining relatively stable over time while patients were on treatment, often irrespective of type of treatment. For instance, an evaluation of amrubicin monotherapy in comparison with carboplatin/etoposide combination therapy as a 1st line treatment in patients aged ≥ 70 years with ED SCLC found that scores for the FACT-L and EQ-5D remained relatively stable over time, with no statistically significant differences either within or between groups (Sekine et al., 2014). However, two trials reported an impact of treatment on global/overall HRQoL. A trend toward a detrimental impact with patients who had undergone prophylactic cranial irradiation (PCI) (vs. observation) (Slotman et al., 2009), and patients taking cisplatin plus etoposide saw a significantly greater increase in HRQoL scores vs. patients taking oral topotecan plus cisplatin (Eckardt et al., 2006).

### Activities of daily living

Sixteen publications presented findings specific to the impact on ADL (Thatcher et al., 2005; O'Brien et al., 2006; Reck et al., 2006, 2012; Chen et al., 2007, 2008; Fennell et al., 2007; Bottomley et al., 2008; Hermes et al., 2008; Rolke et al., 2008; Westerman et al., 2008; Araujo et al., 2009; Lee et al., 2009; Slotman et al., 2009; Le Pechoux et al., 2011; Nagel et al., 2011). The EORTC QLQ-C30 role functioning (RF) domain which assesses limitations in work, daily, and leisure activities and in hobbies, was most often used to asses ADL (*n* = 11). From the publications reviewed, most patients reported some degree of impairment to ADL.

Treatment naïve and chemotherapy/systemic treatment-naïve ED and LD patients' mean baseline scores ranged between 38.7 and 65.6 (Reck et al., 2006; Hermes et al., 2008; Rolke et al., 2008; Westerman et al., 2008; Lee et al., 2009). Studies reporting scores at the high end of this range (62, Westerman et al., 2008 and 65.6, Reck et al., 2006) included both LD and ED patients in their samples, while the lowest scores overall were in systemic treatment naïve ED patients (38.7) (Hermes et al., 2008). Overall ED and LD patient scores differed in the expected direction. LD patients who had a response to treatment reported a score of 74 (Bottomley et al., 2008), while in comparison, ED patients who had responded to treatment had a lower mean score of 65.5 (Slotman et al., 2009). All patient subgroups scored lower than normative data (84.7) (Scott et al., 2008). Two real world studies reported that over two thirds of patients who had completed first line treatment and then relapsed (74.3%, Chen et al., 2008 and 71.6%) (Chen et al., 2007) experienced some degree of interference with daily activities (Chen et al., 2007, 2008). A comparison of LD and ED patients' ADL scores from clinical trials (Reck et al., 2006; Bottomley et al., 2008; Hermes et al., 2008; Lee et al., 2009; Slotman et al., 2009) and real world evidence (Rolke et al., 2008) found similar scores in both types of studies. No publications examined ADL scores by PD1/PD-L1 expression.

Similar to global/overall HRQoL, the majority of publications reported that for patients on treatment the impact on ADL remained stable over time (Giaccone et al., 2005; Bottomley et al., 2008; Hermes et al., 2008; Le Pechoux et al., 2011; Nagel et al., 2011). However, one study highlighted a potential therapeutic benefit of triplet therapy consisting of cisplatin and etoposide plus celecoxib (Fennell et al., 2007), while two studies demonstrated a detrimental impact following treatment with PCI treatment on role functioning for LD and ED SCLC patients (Slotman et al., 2009; Le Pechoux et al., 2011). One publication reported that patients on a combination of Ifosfamide, carboplatin, etoposide, and vincristine had significantly worse impact on ADL at 6 months than those on standard control chemotherapy regimens (Thatcher et al., 2005; Rolke et al., 2008).

### Emotional functioning

Thirteen of the publications reviewed presented findings on EF (Thatcher et al., 2005; Reck et al., 2006, 2012; Fennell et al., 2007; Westerman et al., 2007; Bottomley et al., 2008; Hermes et al., 2008; Rolke et al., 2008; Araujo et al., 2009; Lee et al., 2009; Slotman et al., 2009; Le Pechoux et al., 2011; Nagel et al., 2011). EF was most commonly assessed (*n* = 12) using the four items in the EORTC QLQ-C30 (felt tense, worried, irritable, or depressed). Other PROs assessing EF included the Rotterdam Symptom Checklist (RSCL) or a modified RSCL (*n* = 2) (Thatcher et al., 2005; Fennell et al., 2007) and Hospital Anxiety and Depression Scale (HADS; *n* = 2) (Thatcher et al., 2005; Rolke et al., 2008).

EORTC QLQ-C30 mean baseline scores for ED and LD SCLC patients that had responded to therapy were comparable (80, Slotman et al., 2009 and 77 points, Bottomley et al., 2008, respectively). Both of which were numerically higher but similar to the EORTC QLQ-C30 normative population mean EF score (76.3, Scott et al., 2008). Furthermore, frequency distributions of baseline scores of LD patients in complete remission following treatment revealed that over half of patients had normal levels of EF (59 and 57%) (Le Pechoux et al., 2011). Scores on EF for treatment naïve LD patient scores were comparable to ED patient scores (59.9–65.7, Reck et al., 2006; Hermes et al., 2008; Lee et al., 2009). However, this range was lower than the range of scores of treated patients. In a study comparing groups of different types of lung cancer patients, newly diagnosed treatment naïve SCLC patients experienced higher levels of depression than NSCLC patients (Rolke et al., 2008). A comparison of LD and ED patients' EF scores from clinical trials (Reck et al., 2006; Bottomley et al., 2008; Hermes et al., 2008; Lee et al., 2009; Slotman et al., 2009) and real world evidence (Rolke et al., 2008) found similar scores. No publications examined EF scores by PD1/PD-L1 expression.

Four studies identified a treatment that demonstrated therapeutic superiority in terms of EF (Thatcher et al., 2005; Reck et al., 2006; Hermes et al., 2008; Lee et al., 2009), while five studies showed stability in EF over time (Bottomley et al., 2008; Araujo et al., 2009; Slotman et al., 2009; Le Pechoux et al., 2011; Nagel et al., 2011).

### Cognitive functioning

Thirteen publications reported findings for the impact of SCLC on CF (Reck et al., 2006; Fennell et al., 2007; Bottomley et al., 2008; Hermes et al., 2008; Rolke et al., 2008; Araujo et al., 2009; Lee et al., 2009; Slotman et al., 2009; Kohli et al., 2010; Le Pechoux et al., 2011; Nagel et al., 2011; Wolfson et al., 2011; Nakahara et al., 2015). The only PRO identified in this review assessing CF was the EORTC QLQ-C30, which assesses CF using two items, one of which asks about memory and the other about concentration.

Compared to EORTC QLQ-C30 normative scores of 86.1 (Scott et al., 2008), reported scores and frequency distributions indicate that CF is not necessarily impacted. This was true in treated and untreated LD and ED patients. Comparable or above normative scores were reported for LD (85, Bottomley et al., 2008) and ED (89, Slotman et al., 2009) SCLC patients who had responded to therapy and a sample of treatment naïve LD and ED patients (87, Reck et al., 2006). With the exception of one study (Reck et al., 2006), treatment naïve ED and LD patients' scores were generally lower than the scores of patients who had responded to treatment (ED: 75.3, Hermes et al., 2008 and LD: 76, Lee et al., 2009). Most untreated patients scores were also lower than mean EORTC QLQ-C30 normative scores of 86.1 (Scott et al., 2008). Frequency distributions of baseline scores showed that only 24% of LD SCLC patients in complete remission had below normal CF at baseline (Le Pechoux et al., 2011). The results from real world studies indicates that CF in LD and ED SCLC patients may be impaired with an EORTC QLQ-C30 score of 74.4 (Rolke et al., 2010). This is compared to the range of scores for LD and ED patients' clinical trial mean scores from 75.3 to 89 points (Reck et al., 2006; Bottomley et al., 2008; Hermes et al., 2008; Lee et al., 2009; Slotman et al., 2009). No publications examined CF scores by PD1/PD-L1 expression.

The impact of brain metastases on HRQoL was reported in a single study that investigated the effect of PCI in patients with and without brain metastases. Interestingly, this study reported that neurological deterioration was greater in those patients without brain metastases, compared to patients with brain metastases (Wolfson et al., 2011). The occurrence or development of brain metastases associated with SCLC was reported in seven publications; however no publications reported any effect on CF or any other domains of HRQoL (Eckardt et al., 2009; Le Pechoux et al., 2011; Wolfson et al., 2011; Reck et al., 2012; Satouchi et al., 2014; Sekine et al., 2014; Nakahara et al., 2015).

### Physical functioning

Thirteen publications presented findings for the impact on PF (Reck et al., 2006, 2012; Fennell et al., 2007; Bottomley et al., 2008; Hermes et al., 2008; Rolke et al., 2008; Westerman et al., 2008; Araujo et al., 2009; Lee et al., 2009; Slotman et al., 2009; Le Pechoux et al., 2011; Nagel et al., 2011; Satouchi et al., 2014). The EORTC QLQ-C30 was most commonly used PRO (*n* = 12) to assesses PF, which has five items that ask about the ability to undertake strenuous activities, personal care, and the ability to get around. Additional PROs used to assess PF were the FACT-G (*n* = 1), RSCL (*n* = 1), and QOL-ACD (*n* = 1).

All publications measuring PF in SCLC patients using the EORTC QLQ-C30 reported lower than normative scores (89.8) (Scott et al., 2008). Patients with LD SCLC who had responded to treatment (78) (Bottomley et al., 2008), and a combined sample of treatment naïve LD and ED SCLC patients (77.4) (Reck et al., 2006) reported the highest overall baseline PF scores. Patients with ED SCLC who responded to chemotherapy at trial enrolment had a slightly lower mean score of 66 points (Slotman et al., 2009). Treatment naïve LD and ED patients had EORTC QLQ-C30 comparably lower mean scores (54.7–62) (Hermes et al., 2008; Rolke et al., 2008; Westerman et al., 2008; Lee et al., 2009), with exception to one clinical trial of treatment naïve LD and ED patients (Reck et al., 2006). Treatment naïve ED SCLC patients reported the lowest score overall (54.7) (Hermes et al., 2008). Comparison of real world and clinical trial evidence show that ED and LD real world mean scores are at the low end of the range of LD and ED patients' clinical trial mean scores (56.1, Rolke et al., 2008 vs. 55–78) (Reck et al., 2006; Bottomley et al., 2008; Hermes et al., 2008; Lee et al., 2009; Slotman et al., 2009). No publications examined PF scores by PD1/PD-L1 expression.

Four studies reported that physical functioning either remained stable or increased over time while on treatment, irrespective of type of treatment (Giaccone et al., 2005; Bottomley et al., 2008; Hermes et al., 2008; Satouchi et al., 2014). Two studies reported a potential therapeutic benefit in terms of physical functioning (Reck et al., 2006; Nagel et al., 2011).

### Social functioning

Eleven of the publications reviewed presented findings specific to the impact of SCLC on SF (Reck et al., 2006, 2012; Fennell et al., 2007; Bottomley et al., 2008; Hermes et al., 2008; Rolke et al., 2008; Araujo et al., 2009; Lee et al., 2009; Slotman et al., 2009; Le Pechoux et al., 2011; Nagel et al., 2011). Most studies (*n* = 9) used the EORTC QLQ-C30, which assesses SF using a single item that examines interference with family life and with social activities. One study used the Functional Assessment Cancer-Therapy General (FACT-G) to assess social well-being (SWB) (Reck et al., 2006).

In comparison to normative scores (87.5, Scott et al., 2008), LD and ED treated and treatment naïve patients are impacted by SCLC. Treated ED patients and LD patients who have undergone at least first line treatment and responded to therapy (74.5, Slotman et al., 2009 and 74, Bottomley et al., 2008, respectively) were least impacted of all groups. Similar baseline scores were reported between ED SCLC and LD SCLC patients who were already treated. Scores in SCLC ED and LD patients who were treatment/chemotherapy naïve ranged from 58.5 (Hermes et al., 2008) to 72.5 (Reck et al., 2006). While the scores ranged considerably, no difference in scores was found between combined samples of treatment naïve LD and ED patients (Reck et al., 2006; Rolke et al., 2008; Lee et al., 2009) and treatment naïve ED patients (Hermes et al., 2008). A study reported that 58% of LD SCLC patients who were in complete remission following treatment have below normal SF scores (Le Pechoux et al., 2011). Similar to PF, the SF mean score of LD and ED patients taking part in a real world study was at the low end of the range of LD and ED patients' clinical trial scores (58.5, Rolke et al., 2008 vs. 58.3–74.5 Reck et al., 2006; Bottomley et al., 2008; Hermes et al., 2008; Lee et al., 2009; Slotman et al., 2009). No publications examined SF scores by PD1/PD-L1 expression.

SF was usually shown to be consistent over time. However, some impact was demonstrated with treatment, with one author reporting statistically significant improvements in both LD and ED SCLC patients following combination treatment of chemotherapy and Darbepoetin alfa (Nagel et al., 2011). Two publications reported on the effect of PCI in SCLC patients, with one study identifying an improvement in the social functioning of LD SCLC patients over time while on treatment (*p* = 0.009) (Le Pechoux et al., 2011) and the other study reported a decline in social function of ED SCLC patients (*p* < 0.05) (Slotman et al., 2009).

## Discussion

This review identified that the negative impact of SCLC extends beyond overall HRQoL to most of the domains included within HRQoL. Further, there was evidence to suggest that the impact was greatest in treatment naive ED patients. This is similar with previous findings in NSCLC that the impact on HRQoL is greater in later stage cancer (Enstone et al., 2015). The impact was generally least in both LD and ED patients that had responded to treatment. The only LD SCLC-exclusive patient samples included in the review had been treated and responded to therapy, while conversely ED patients were generally treatment/chemotherapy naïve. No scores were available pertaining to treatment naïve LD patients. Scores from this population of SCLC patients would provide a more comprehensive understanding of the impact of disease stage on HRQoL.

While global or overall HRQoL scores were most commonly reported, there was some evidence to indicate that the impact of SCLC may be greatest in the domains of ADL and PF, and the least impact on EF and CF domains. However, this review identified that there was a lack of reporting of domain level scores to allow for a more granular level of understanding from which to draw robust conclusions. Therefore, future research should report all domain level results as well as overall or global HRQoL.

There were some inconsistencies in the magnitude of impact. Due to the heterogeneity of results it is unclear if there was an impact in the CF and EF domains. The inconsistency in the CF domain mirrors that found in NSCLC patients (Enstone et al., 2015). The difference in magnitude may be explained by several factors. Studies involving treatment naïve and ED patients generally reported lower HRQoL domain scores, however the majority of studies did not elaborate between these subgroups and other groups, therefore the impact is diluted. There were instances, where a combined sample of treatment naïve LD and ED patients reported comparable scores to treated patients, which were generally higher than treatment naïve patient scores (Reck et al., 2006). It was clear from this review that very few publications presented data by subpopulations of SCLC patients. Many presented the results of combined LD and ED samples and one study only demonstrated that smoking negatively impacted HRQoL for SCLC patients (Chen et al., 2012). Furthermore, despite the fact that about 40–50% of those with SCLC develop brain metastases (Quan et al., 2004), only one paper presented HRQoL data for this specific subpopulation (Wolfson et al., 2011). Therefore, there is no conclusive empirical evidence to inform whether the presence or absence of brain metastases impairs cognitive function, or the other domains of HRQoL. Additionally, while only three of all publications reviewed reported including PD1/PD-L1 expression in their sample population, no subgroup analyses were reported in relation to HRQoL. From the remainder of the articles included in this review, no mention was made of PD1/PD-L1 expression. In comparison to NSCLC, PD-L1 is currently of less importance to SCLC, as PD-L1 expression has yet to predict treatment benefit in SCLC (Antonia et al., 2016).

A key objective of our study was to identify the PROs being used to measure HRQoL for SCLC patients. The most frequently used PRO to assess HRQoL was the EORTC QLQ-C30 core cancer instruments, the lung cancer specific module the EORTC QLQ-LC13, LCSS, and EQ-5D. The EORTC instruments, while considered by some authors as the standard for HRQoL measurement in lung cancer (Koller et al., 2015), were developed prior regulatory PRO guidance. The LCSS appears to be face and content valid for this patient population (Hollen et al., 1993). Although the EORTC instruments have been included in European summary of product characteristics (SmPC), an FDA labeling claim has yet to be obtained. The lack of opportunity for obtain a labeling claim by using these instruments may play a part in why HRQoL and specific domains of HRQoL are not receiving significant focus in the literature, despite their frequent use as a secondary endpoint in clinical trials. These instruments are currently being updated; however it is unclear if this is in consideration of FDA guidance (Koller et al., 2015).

This study was limited due to the availability of published HRQoL scores and findings. This extends to a lack of sub-population analysis and reporting of domain level scores and findings. Optimally a meta-analysis of scores would have been conducted. However, despite the wide use of the EORTC QLQ-C30 indicating some standardization of data collection, there was an uneven reporting of actual scores and domain-level data as well as heterogeneity in the study design and study populations. Thus, a meta-analysis of HRQoL data would have yielded unreliable and invalid results.

While the inclusion of HRQoL outcomes as endpoints in clinical trials and real world studies of SCLC is becoming more common, this review found limitations in the way these HRQoL data have been reported, specifically the domain-level data. A review of literature summarizing the use of the EORTC QLQ-LC13 in clinical trials supports this finding, reporting that of the 109 trials found to have included the instrument, 83 reported results and only 69 publications (63%) provided numerical values (Koller et al., 2015). The lack of published HRQoL data may be due to a publication bias, with researchers not publishing findings in cases where the data does not present favorable outcomes (decline in HRQoL) in relation to the treatment being investigated. Initiatives such as COMET have attempted to ensure that key endpoints, including those of importance to patients such as HRQoL, are always reported (Initiative, 2016) however, this is not yet commonplace.

It is clear from this review that there are important gaps in the knowledge base which need to be addressed in order to make meaningful conclusions about HRQoL in SCLC that can be applied to treatment decisions. Payers and decision makers are increasingly using HRQoL data to make reimbursement and prescribing decisions. As healthcare systems are increasingly pushed to their financial limits and require evidence of value for reimbursement decisions, the importance of collection and publication of patient-centered outcomes such as HRQoL increases. In future research it is important that all HRQoL data, including domain-level and subpopulation analyses to enable the most informed decisions to be made regarding treatment for SCLC patients.

## Author contributions

All authors contributed to the study concept, design, manuscript editing and reviewing. BB, JW, and CP were responsible for data acquisition, analysis and interpretation, and manuscript preparation.

### Conflict of interest statement

BB, JW, and CP work at Adelphi Values who were employed by BMS as expert consultants to carry out the review described in this manuscript. YY and JP are employees of BMS who funded this work.

## References

[B1] Antonia S. J. López-Martin J. A. Bendell J. Ott P. A. Taylor M. Eder J. P.. (2016). Nivolumab alone and nivolumab plus ipilimumab in recurrent small-cell lung cancer (CheckMate 032): a multicentre, open-label, phase 1/2 trial. Lancet Oncol. 17, 883–895. 10.1016/S1470-2045(16)30098-5 27269741

[B2] Araujo A. M. Mendez J. C. Coelho A. L. Sousa B. Barata F. Figueiredo A. (2009). Phase II study of celecoxib with cisplatin plus etoposide in extensive-stage small cell lung cancer. Cancer Invest. 27, 391–396. 10.1080/07357900802232756 19266367

[B3] Bottomley A. Debruyne C. Felip E. Millward M. Thiberville L. D'Addario G.. (2008). Symptom and quality of life results of an international randomised phase III study of adjuvant vaccination with Bec2/BCG in responding patients with limited disease small-cell lung cancer. Eur. J. Cancer 44, 2178–2184. 10.1016/j.ejca.2008.06.036 18676140

[B4] Chen J. Qi Y. Wampfler J. A. Jatoi A. Garces Y. I. Busta A. J.. (2012). Effect of cigarette smoking on quality of life in small cell lung cancer patients. Eur. J. Cancer 48, 1593–1601. 10.1016/j.ejca.2011.12.002 22244802 PMC3404819

[B5] Chen L. Antras L. Duh M. S. Levy N. Neary M. O'Brien M. E.. (2007). Psychometric validation of the patient symptom assessment in lung cancer instrument for small cell lung cancer. Curr. Med. Res. Opin. 23, 2741–2752. 10.1185/030079907X233331 17900394

[B6] Chen L. Antras L. Duh M. S. Neary M. O'Brien M. E. (2008). Symptom assessment in relapsed small cell lung cancer: cross-validation of the patient symptom assessment in lung cancer instrument. J. Thorac. Oncol. 3, 1137–1145. 10.1097/JTO.0b013e3181861729 18827610

[B7] Eckardt J. R. Bentsion D. L. Lipatov O. N. Polyakov I. S. Mackintosh F. R. Karlin D. A.. (2009). Phase II study of picoplatin as second-line therapy for patients with small-cell lung cancer. J. Clin. Oncol. 27, 2046–2051. 10.1200/JCO.2008.19.3235 19289620

[B8] Eckardt J. R. von Pawel J. Papai Z. Tomova A. Tzekova V. Crofts T. E.. (2006). Open-label, multicenter, randomized, phase III study comparing oral topotecan/cisplatin versus etoposide/cisplatin as treatment for chemotherapy-naive patients with extensive-disease small-cell lung cancer. J. Clin. Oncol. 24, 2044–2051. 10.1200/JCO.2005.03.3332 16648504

[B9] Enstone A. Panter C. Manley Daumont M. Miles R. (2015). Societal burden and impact on health related quality of life (HRQoL) of non-small cell lung cancer (NSCLC), in 18th Annual ISPOR (Milan), 2015.

[B10] Fennell D. A. Steele J. P. Shamash J. Slater S. E. Sheaff M. T. Wells P.. (2007). Phase II trial of irinotecan, cisplatin and mitomycin for relapsed small cell lung cancer. Int. J. Cancer 121, 2575–2577. 10.1002/ijc.22984 17680556

[B11] Giaccone G. Debruyne C. Felip E. Chapman P. B. Grant S. C. Millward M.. (2005). Phase III study of adjuvant vaccination with Bec2/bacille Calmette-Guerin in responding patients with limited-disease small-cell lung cancer (European Organisation for Research and Treatment of Cancer 08971-08971B; Silva Study). J. Clin. Oncol. 23, 6854–6864. 10.1200/JCO.2005.17.186 16192577

[B12] Gorman G. (2012). New and emerging strategies for the treatment of small cell lung cancer. J. Pharmaceut. Sci. Emerg. Drugs 1, 1–2. 10.4172/2380-9477.1000e101

[B13] Henry D. H. Viswanathan H. N. Elkin E. P. Traina S. Wade S. Cella D. (2008). Symptoms and treatment burden associated with cancer treatment: results from a cross-sectional national survey in the US. Support. Care Cancer 16, 791–801. 10.1007/s00520-007-0380-2 18204940

[B14] Hermes A. Bergman B. Bremnes R. Ek L. Fluge S. Sederholm C.. (2008). Irinotecan plus carboplatin versus oral etoposide plus carboplatin in extensive small-cell lung cancer: a randomized phase III trial. J. Clin. Oncol. 26, 4261–4267. 10.1200/JCO.2007.15.7545 18779613

[B15] Higgins J. Green S. (2005). Cochrane Handbook for Systematic Reviews of Interventions 4.2. 5 [Updated May 2005]. Chichester: The cochrane library.

[B16] Hollen P. J. Gralla R. J. Kris M. G. Potanovich L. M. (1993). Quality of life assessment in individuals with lung cancer: testing the Lung Cancer Symptom Scale (LCSS). Eur. J. Cancer 29, S51–S58. 10.1016/s0959-8049(05)80262-x 8381294

[B17] Initiative C. (2016). Core Outcomes Measures in Effectiveness Trials [Online]. Available online at: http://www.comet-initiative.org/ (Accessed October 14, 2016).

[B18] Jatoi A. Williams B. A. Marks R. Nichols F. C. Aubry M. C. Wampfler J. Yang P. (2005). Exploring vitamin and mineral supplementation and purported clinical effects in patients with small cell lung cancer: results from the mayo clinic lung cancer cohort. Nutr. Cancer 51, 7–12. 10.1207/s15327914nc5101_2 15749624

[B19] Kohli S. Novotny P. J. Sloan J. A. Buckner J. C. Brown P. D. Yang P. (2010). Cancer treatment impact on cognitive functioning and quality of life in lung cancer survivors. J. Clin. Oncol. 28(15 Suppl.), 9112 10.1200/jco.2010.28.15_suppl.9112

[B20] Koller M. Warncke S. Hjermstad M. J. Arraras J. Pompili C. Harle A.. (2015). Use of the lung cancer–specific Quality of Life Questionnaire EORTC QLQ-LC13 in clinical trials: a systematic review of the literature 20 years after its development. Cancer 121, 4300–4323. 10.1002/cncr.29682 26451520

[B21] Le Pechoux C. Laplanche A. Faivre-Finn C. Ciuleanu T. Wanders R. Lerouge D.. (2011). Clinical neurological outcome and quality of life among patients with limited small-cell cancer treated with two different doses of prophylactic cranial irradiation in the intergroup phase III trial (PCI99-01, EORTC 22003-08004, RTOG 0212 and IFCT 99-01). Ann. Oncol. 22, 1154–1163. 10.1093/annonc/mdq576 21139020 PMC3082159

[B22] Lee S. M. Woll P. J. Rudd R. Ferry D. O'Brien M. Middleton G.. (2009). Anti-angiogenic therapy using thalidomide combined with chemotherapy in small cell lung cancer: a randomized, double-blind, placebo-controlled trial. J. Natl. Cancer Inst. 101, 1049–1057. 10.1093/jnci/djp200 19608997

[B23] Nagel S. Kellner O. Engel-Riedel W. Guetz S. Schumann C. Gieseler F.. (2011). Addition of darbepoetin alfa to dose-dense chemotherapy: results from a randomized phase II trial in small-cell lung cancer patients receiving carboplatin plus etoposide. Clin. Lung Cancer 12, 62–69. 10.3816/CLC.2011.n.009 21273182

[B24] Nakahara Y. Takagi Y. Okuma Y. Hosomi Y. Okamura T. Shibuya M.. (2015). Neurotoxicity due to prophylactic cranial irradiation for small-cell lung cancer: a retrospective analysis. Mol. Clin. Oncol. 3, 1048–1052. 10.3892/mco.2015.581 26623048 PMC4535021

[B25] O'Brien M. E. Ciuleanu T. E. Tsekov H. Shparyk Y. Cucevia B. Juhasz G.. (2006). Phase III trial comparing supportive care alone with supportive care with oral topotecan in patients with relapsed small-cell lung cancer. J. Clin. Oncol. 24, 5441–5447. 10.1200/JCO.2006.06.5821 17135646

[B26] Quan A. L. Videtic G. M. Suh J. H. (2004). Brain metastases in small cell lung cancer. Oncology 18, 961–972. 15328892

[B27] Reck M. Thatcher N. Smit E. F. Lorigan P. Szutowicz-Zielinska E. Liepa A. M.. (2012). Baseline quality of life and performance status as prognostic factors in patients with extensive-stage disease small cell lung cancer treated with pemetrexed plus carboplatin vs. etoposide plus carboplatin. Lung Cancer 78, 276–281. 10.1016/j.lungcan.2012.09.002 23043970

[B28] Reck M. von Pawel J. Macha H. N. Kaukel E. Deppermann K. M. Bonnet R.. (2006). Efficient palliation in patients with small-cell lung cancer by a combination of paclitaxel, etoposide and carboplatin: quality of life and 6-years'-follow-up results from a randomised phase III trial. Lung Cancer 53, 67–75. 10.1016/j.lungcan.2006.04.001 16713013

[B29] Rolke H. B. Bakke P. S. Gallefoss F. (2008). Health related quality of life, mood disorders and coping abilities in an unselected sample of patients with primary lung cancer. Respir. Med. 102, 1460–1467. 10.1016/j.rmed.2008.04.002 18590954

[B30] Rolke H. B. Bakke P. S. Gallefoss F. (2010). HRQoL changes, mood disorders and satisfaction after treatment in an unselected population of patients with lung cancer. Clin. Respir. J. 4, 168–175. 10.1111/j.1752-699X.2009.00171.x 20565496

[B31] Satouchi M. Kotani Y. Shibata T. Ando M. Nakagawa K. Yamamoto N.. (2014). Phase III study comparing amrubicin plus cisplatin with irinotecan plus cisplatin in the treatment of extensive-disease small-cell lung cancer: JCOG 0509. J. Clin. Oncol. 32, 1262–1268. 10.1200/JCO.2013.53.5153 24638015

[B32] Scott N. Fayers P. Aaronson N. Bottomley A. de Graeff A. Groenvold M. (2008). EORTC QLQ-C30 References Values Reference Values. Brussels: EORTC.

[B33] Sekine I. Okamoto H. Horai T. Nakagawa K. Ohmatsu H. Yokoyama A.. (2014). A randomized phase III study of single-agent amrubicin vs. carboplatin/etoposide in elderly patients with extensive-disease small-cell lung cancer. Clin. Lung Cancer 15, 96–102. 10.1016/j.cllc.2013.11.006 24361248

[B34] Siegel R. L. Miller K. D. Jemal A. (2015). Cancer statistics. CA Cancer J. Clin. 65, 5–29. 10.3322/caac.21254 25559415

[B35] Slotman B. J. Mauer M. E. Bottomley A. Faivre-Finn C. Kramer G. W. Rankin E. M.. (2009). Prophylactic cranial irradiation in extensive disease small-cell lung cancer: short-term health-related quality of life and patient reported symptoms: results of an international Phase III randomized controlled trial by the EORTC Radiation Oncology and Lung Cancer Groups. J. Clin. Oncol. 27, 78–84. 10.1200/JCO.2008.17.0746 19047288 PMC2645093

[B36] Thatcher N. Qian W. Clark P. I. Hopwood P. Sambrook R. J. Owens R.. (2005). Ifosfamide, carboplatin, and etoposide with midcycle vincristine versus standard chemotherapy in patients with small-cell lung cancer and good performance status: clinical and quality-of-life results of the British Medical Research Council multicenter randomized LU21 trial. J. Clin. Oncol. 23, 8371–8379. 10.1200/JCO.2004.00.9969 16293867

[B37] Westerman M. J. Hak T. Sprangers M. A. Groen H. J. van der Wal G. The A. M. (2008). Listen to their answers! Response behaviour in the measurement of physical and role functioning. Qual. Life Res. 17, 549–558. 10.1007/s11136-008-9333-6 18389384 PMC2358935

[B38] Westerman M. J. The A. M. Sprangers M. A. Groen H. J. van der Wal G. Hak T. (2007). Small-cell lung cancer patients are just ‘a little bit’ tired: response shift and self-presentation in the measurement of fatigue. Qual. Life Res. 16, 853–861. 10.1007/s11136-007-9178-4 17450423 PMC1915653

[B39] Wolfson A. H. Bae K. Komaki R. Meyers C. Movsas B. Le Pechoux C.. (2011). Primary analysis of a phase II randomized trial Radiation Therapy Oncology Group (RTOG) 0212: impact of different total doses and schedules of prophylactic cranial irradiation on chronic neurotoxicity and quality of life for patients with limited-disease small-cell lung cancer. Int. J. Radiat. Oncol. Biol. Phys. 81, 77–84. 10.1016/j.ijrobp.2010.05.013 20800380 PMC3024447

[B40] World Health Organization (2012). Lung Cancer Estimated Incidence, Mortality and Prevalence Worldwide in 2012 [Online]. Available online at: http://globocan.iarc.fr/Pages/fact_sheets_cancer.aspx?cancer=lung (Accessed October, 2016).

[B41] Zikos E. Ghislain I. Coens C. Ediebah D. E. Sloan E. Quinten C.. (2014). Health-related quality of life in small-cell lung cancer: a systematic review on reporting of methods and clinical issues in randomised controlled trials. Lancet Oncol. 15, e78–e89. 10.1016/S1470-2045(13)70493-5 24480558

